# The Thromboembolic Risk in Giant Cell Arteritis: A Critical Review of the Literature

**DOI:** 10.1155/2014/806402

**Published:** 2014-05-20

**Authors:** A. Guida, A. Tufano, P. Perna, P. Moscato, M. T. De Donato, R. Finelli, D. Caputo, M. N. D. Di Minno

**Affiliations:** ^1^U.O.C. Medicina d'Urgenza, A.O.U. “S. Giovanni e Ruggi”, Largo Città di Ippocrate 1, 84100 Salerno, Italy; ^2^Regional Reference Centre for Coagulation Disorders, University “Federico II”, Naples, Italy; ^3^U.O.C. Medicina Generale, A.O.U. “S. Giovanni e Ruggi”, Largo Città di Ippocrate 1, 84100 Salerno, Italy

## Abstract

Giant cell arteritis is a systemic vasculitis characterized by granulomatous inflammation of the aorta and its main vessels. Cardiovascular risk, both for arterial and venous thromboembolism, is increased in these patients, but the role of thromboprophylaxis is still debated. It should be suspected in elderly patients suffering from sudden onset severe headaches, jaw claudication, and visual disease. Early diagnosis is necessary because prognosis depends on the timeliness of treatment: this kind of arteritis can be complicated by vision loss and cerebrovascular strokes. Corticosteroids remain the cornerstone of the pharmacological treatment of GCA. Aspirin seems to be effective in cardiovascular prevention, while the use of anticoagulant therapy is controversial. Association with other rheumatological disease, particularly with polymyalgia rheumatica is well known, while possible association with antiphospholipid syndrome is not established. Large future trials may provide information about the optimal therapy. Other approaches with new drugs, such as TNF-alpha blockades, Il-6 and IL-1 blockade agents, need to be tested in larger trials.

## 1. Introduction

Vasculitis syndromes are inflammatory diseases affecting large-, medium-, or small-sized vessels, caused by various immunological processes and possibly triggered by infectious agents [1]. Thromboembolic disease is an increasing complication of several and rheumatic diseases. In Behcet's disease, thrombosis is a common clinical feature, while its role in ANCA associated vasculitis is emerging [2]. Temporal arteritis, also known as cranial arteritis or giant cell arteritis (GCA), is a chronic systemic inflammation of the medium- and large-size arteries characterized by granulomatous lesions; typically, it concerns one or more branches of the carotid artery, especially the temporal artery, involving aortic arch, axillary, iliac, and the femoral arteries as well [3]. Both venous and arterial events have been described in this setting; several case series of myocardial infarction and stroke have been reported [4]. Rare in individuals younger than 50 years, the peak of disease onset is in the 7th decade; female/male ratio is 3 : 1 [5].

The incidence varies from 10 to 29 cases per 100.000 inhabitants per year in the United States [6]; for GCA the prevalence reported is 8–10% people per 100.000 inhabitants, with a peak of 200 cases/100.000 inhabitants, over 50 years of age [7]; a higher incidence is reported in extreme northern latitudes [8]; it is rare among the Afro-American population [9]; familial cases have been reported [10]. In 50–60% of patients, GCA is associated with the polymyalgia rheumatica (PR). An increased cardiovascular and thromboembolic risk in vasculitis is largely reported and seems to be related to the period of higher activity of the diseases [1, 3]. The role of traditional risk factor for cerebrovascular ischemic event in this setting is still debated; a retrospective Italian study [11] reported the following as major risk factors for ischemic stroke: arterial hypertension and previous ischemic heart disease, while systemic clinical manifestation and high RCP levels were protective.

## 2. Clinical Characteristics and Diagnosis

Severe headache is probably the most common onset symptom and is present in about two-thirds of the patients; scalp tenderness is usually limited to the temporal arteries, but it may also involve larger areas. The arteries involved appear thickened, nodulous, painful, and erythematous with impaired or absent pulse (see Table 1).

Almost half of the patients present “jaw claudication” tongue pain and impaired swallowing; seldom a severe vascular thickening can lead to scalp infarction or tongue infarction. Permanent visual loss, partial or total, occurs in up to 20% of the patients and is often the first manifestation of the disease [12]. Once established, the visual deficiency is usually permanent.* Amaurosis fugax* precedes permanent loss in 44% of the patients. Visual loss/deficiency is caused by ischemia of the nerve or of the optic tract as a result of arteritis of the branches of the posterior ciliary artery or of the ophthalmic artery and, less commonly, occlusion of the retinal arterioles, causing ischemic optic neuritis with slight paleness and oedema of the optic disk, some cotton-like exudates, and small hemorrhages [13]. Atherosclerotic risk factor may influence cardiovascular risk in GCA patients. In a series of 210 GCA patients presenting with classic cardiovascular risk factors, the OR for at least a major ischemic event was 1.79 (95% CI 1.03–3.11; *P* = 0.05). Among GCA patients with arterial hypertension this risk was significantly increased (OR, 1.80; 95% CI, 1.00–3.25; *P* = 0.05). The study suggests that the presence of atherosclerosis risk factors at the time of diagnosis of GCA may influence the development of severe ischemic manifestations of the disease [14]. A recent large cohort study [15] on 3408 GCA patients evaluated cardiovascular risk (CV) in GCA patients as compared to a sample of controls and found an increased incidence of CV events (myocardial infarction, peripheral arterial disease, and cerebrovascular accident) with an incidence rate ratio of 1.68 (95% CI 1.49–1.89).

Interestingly the association with cardiovascular disease is stronger in the first month after diagnosis with a hazard ratio (HR) of 4.92 (95% CI 2.59–9.34) and 1.70 (95% CI 1.51–1.91) during the follow-up period.

About 30% of the patients present neurological manifestations [16]; the most common are neuropathies (14%), including mono- and polyneuropathies of the limbs; stroke has been extensively described, particularly vertebrobasilar ischemia, and the rate of stroke/transient ischemic attack (TIA) reported is 5–20%; the mechanism underlying is related to the atherosclerosis due to the older age of patients and to the endothelial dysfunction that characterize GCA. Gonzalez-Gay et al. reported a 2.8% incidence of ischemic stroke within the 4th week since diagnosis [17]; the majority of strokes occurred in the vertebrobasilar artery territory; in this case series arterial hypertension, male sex, and smoke were strongly associated with ischemic cerebral disease; female sex and anemia were protective factors; the most predictive factor for vertebrobasilar occlusion was history of smoking.

The strongest risk factor for overall ischemic cerebral events remains the monocular visual loss; the lesions are often of embolic origin [17, 18]. This report underlines the importance of traditional cardiovascular risk factor in determining cerebrovascular events in this setting of patients and highlights the impact of visual loss as predictive factor of ischemic stroke.

A rare cause of stroke (less than 1%) is represented by cerebral vein thrombosis (CVT). Donadini et al. [19] described an interesting case of cerebral vein thrombosis in a 63-year-old woman presenting with left migraine, monocular visual loss, and absence of the ipsilateral temporal pulse. MRI detected the left sigmoid sinus and ipsilateral jugular vein thrombosis while temporal artery biopsy was necessary for GCA diagnosis.

Involvement of intracranial arteries is rare and fatal, often described in postmortem studies, because of the severity of disease presenting with these features; the majority of patients with intracranial vasculitis had a worse prognosis and an extensive cerebral damage with poor response to immunosuppressive therapy, and this finding was associated with a catastrophic course of disease, and the rare cases presenting only angiographic intracranial defects, without symptoms, had a better prognosis and response to therapy [20]. Concluding, the majority of cerebrovascular ischemic events are due to the involvement of internal carotid or vertebrobasilar arteries, while arteritis of intracranial district is rare and limited to a subset of GCA patients with a poor prognosis and a worse outcome with corticosteroids therapy.

A vestibular dysfunction or a hearing deficiency can be present [21]. Many authors consider the polymyalgia rheumatica and GCA as different stages of the same disease; about 16–21% of the patients with polymyalgia rheumatica also present giant cell arteritis, while 40–60% of the patients with GCA have polymyalgia rheumatica [22]. The coronary artery involvement in GCA is almost rare but significantly associated with myocardial infarction (MI), as described in several case series, and MI has been reported even during corticosteroid therapy [23]; ischemic heart disease is a negative prognostic factor in GCA. As reported by Gonzalez-Gay and coworkers [24] mortality of GCA subjects (as standardized mortality ratio) (SMR) with coronary artery disease was higher than general population, 1.62(95% CI 0.7–3.20), and was higher even when compared to those GCA subjects who did not experience myocardial events, with a hazard ratio of 3.42 (95% CI 1.85–6.33); *P* = 0.0001. Biopsy proven GCA with coronary involvement represents a risk factor for aortic aneurism/dissection and/or large artery stenosis with an odds ratio (OR) of 3.88 (95% CI 1.49–10.21; *P* = 0.004) particularly in those patients with arterial hypertension [25].

The thoracic aortic aneurysm is 17 times more frequent in patients affected by GCA [26], while abdominal aortic aneurism is 2.7 times likely to be detected [26]; the incidence of large artery stenosis in GCA ranged from 18–21% for arm arteries to 9–14% for any large artery complication. Nuenninghoff and coworkers [27] conducted a retrospective cohort study on 168 GCA patients to determine the predictive factors of aortic aneurism, aortic dissection, and large artery stenosis in GCA; 27% of subjects presented large vessel involvement (aortic aneurysm/dissection, large vessel stenosis); hyperlipidemia and coronary artery diseases were associated with aortic aneurysm and/or dissection (*P* < 0.05 for both), while cranial symptoms and elevated ESR were negatively associated with large artery stenosis, previous TIA or stroke, and/or diminished pulse or blood pressure and were predictors of these complications. According to Spanish study results, arterial hypertension and marked inflammatory disease were strictly predictive of aortic aneurism development [28, 29].

Rarely, both polymyalgia rheumatica and Horton's arteritis are associated with lung neoplasm or hematological disorders (i.e., macroglobulinemia of Waldenstrom) [30].

### 2.1. Diagnosis

A high percentage (40%) of patients present nonspecific symptoms. Except for histopathology of the artery wall, neither laboratory data nor specific signs or symptoms exist for the diagnosis of temporal arteritis. The classification criteria proposed by the American College of Rheumatology are currently used (Table 2).

For each of these criteria, the presence of all the characteristics is needed in order to formulate the diagnosis of GCA. The presence of three or more criteria leads to a 93.5% sensibility and to a 91.2% specificity. The superficial temporal artery biopsy represents the gold standard for the diagnosis of giant cell arteritis [31, 32], while a negative result does not exclude it; 10% to 15% of the biopsies are falsely negative. When the extracranial arteries seem normal to the palpation and a giant cell arteritis is suspected and when clinical signs are vague, a long segment of the temporal artery (from 3 to 5 cm) is needed and a counterlateral biopsy could be necessary if the first results are normal.

The ultrasound of the temporal artery is considered to be helpful in the diagnosis. In the ultrasound color-doppler, a “halo” effect around the temporal artery is a specific aspect in patients with clinically active arteritis and confirms the diagnosis; however its sensibility is low (40%); therefore the absence of this sign does not exclude it [33]. If an extracranial involvement of giant cell arteritis is suspected, an arteriography, a computed tomography (CT) scan, and a magnetic resonance (MRI) are required as diagnostic tests. In the arteriography, the typical aspect is the stenosis or the bilateral occlusion of the subclavian, the axillary, and the proximal brachial arteries that appear homogenous and thinned [34, 35]. Intracranial artery involvement is rare and when symptomatic predicts a worse prognosis [20].

### 2.2. Pathogenesis

Polymyalgia rheumatica and GCA represent polygenic disorders in which multiple genetic and environmental factors are involved, determining their severity. In GCA inflammation involves all vassal wall strata, with mononucleate infiltrate in the vasal wall and presence of giant cells. The cellular branch of the adaptive and innate immune systems appears to be central to the pathogenesis of GCA; autoreactive CD4-positive T lymphocytes, including INF-*γ* producing T-helper (Th) 1 cells and IL-17-secreting Th17 cells, orchestrate the development of granulomatous vascular inflammation. The T-regulator cells Fox3+, which normally limit immune response, are reduced while rising IL-6, a pleiotropic cytokine, produced by T cell, B cell, macrophages, and fibroblasts. The endothelial cells participate in the activation of T cells, the terminal differentiation of B cells, the survival of plasmocytes, the differentiation of Th17 lymphocytes, and the inhibition of Treg-cell differentiation and function. Thus, the IL-6 pathway represents the intersection of the innate and acquired immune systems and, when unregulated, maintains inflammation both in polymyalgia rheumatica and in GCA [1, 36, 37]. The HLA-DRB1*04 and -DRB1*01 alleles are associated with a greater susceptibility and severity of both diseases [38]. Viral antigens (parvovirus, influenza virus, herpes zoster virus, and* Chlamydia pneumonia*), toxins, drugs, and autoantigens of the arteries can spark the immune reaction [39, 40]. The macrophages produce in the adventitia inflammatory cytokines such as interleukin-1 (IL-1) and interleukin-6 (IL-6), while in the media and intima they contribute to the vascular damage elaborating metalloproteinases and nitric oxide, respectively. This mechanism is counterbalanced from fibrosis and angiogenesis, mediated by platelet derived growth factors (PDGF) and vascular endothelium growth factor (VEGF), secreted by mononuclear and multinuclear giant cells [41]. The final result is an occlusive vasculopathy caused by the rapid proliferation of the* intima*. Patients with ischemic symptoms have a higher concentration of mRNA coding for gamma interferon (*γ*-INF) and for IL-1b [42], suggesting that production of *γ*-INF is crucial for the development of vasculitis, particularly for the vascular occlusion, directly associated with the presence of *γ*-INF* in situ* [5].

Thromboembolic diseases, both arterial and venous thrombosis, have been described in several vasculitis [43]; the link between haemostatic system and vascular damage seems to be the inflammation. The key role of this mechanism concerns cytokines such as IL-1, tumor necrosis factor alpha (TNF-*α*), and IL-6. Endothelial cells release adhesion molecules (TXA2, VCAM-1, I-CAM-1, P-selectin, and E-selectin) and procoagulant factors (vWF, TF) promoting coagulation cascade and leukocytes trafficking. Increased TXA2 activates platelets that, together with the increase in tissue factor (TF)/FVII interaction, promote the coagulation cascade. Not only hypercoagulability but also anticoagulant protein reduction contributes to thrombosis in this setting: protein C is decreased for consumption; TF pathway inhibitor (TFPI) production is impaired and is also rapidly consumed, while fibrinolysis is compromised because of endothelial PAI-1 hyperproduction stimulated by IL-1 and TNF-*α* [4, 5, 18]; Vrij et coll. [44] described an antithrombin levels reduction with a concomitant TAT increase in active GCA versus inactive GCA, hypothesizing a consumption due to a disseminated intravascular coagulation during the active phase of the disease. D-Dimer has been largely described as a marker of coagulation activity in several rheumatic diseases [45], but no peculiar role has been identified in GCA. The impact of the thrombophilic conditions (anticoagulant proteins deficiency, antiphospholipid syndrome, factor V Leiden, and G20210a polymorphism of prothrombin gene) in GCA patients was evaluated and no association emerged [46]; association within antiphospholipid antibodies syndrome and GCA remains so far anecdotal and a linkage between antifospholipids syndrome and thrombosis, in this setting, seems to be not statistically significant [47, 48].

### 2.3. Therapy and Thromboprophylaxis

Antithrombotic therapy is an important part of GCA therapy. In Behcet's disease immunosuppressive therapy is effective in preventing thrombotic complications. In experimental models, aspirin (ASA) decreases the transcriptional activation of the *γ*-INF gene, as a consequence, inhibiting a key point in the pathogenesis of the disease [49]. Low dose (75–100 mg) aspirin is successfully prescribed in cardiovascular prevention in GCA [50] and other autoimmune diseases, even if many authors did not agree with this statement. Salvarani et al. [11] reported nonefficacy of antiplatelet/anticoagulant therapy in risk reduction of cranial vascular disease; this may be because in this cohort most of patients on antithrombotic therapy already experienced ischemic events; for this reason they presented a higher intrinsic risk. Gonzalez-Gay and coworkers [17] did not observe any effect on the risk of severe ischemic complication. Berger and coworkers did not find a protective effect of aspirin therapy in a GCA retrospective cohort, while a strong association between jaw claudication and cardiovascular event was reported. Platelet number and platelet size were also considered and were not predictive of ischemic events [51].

Venous thromboembolism (VTE) is reported as case series in GCA; by the way, thromboprophylaxis in high-risk conditions such as surgery, immobilization, and acute medical illness should be considered. A possible link between VTE and GCA is due to coagulation cascade activation due to systemic inflammation; for this reason a thromboprophylaxis in particular situations may be considered.

Corticosteroids are the therapy of choice to treat giant cell arteritis, usually lasting 2-3 years [52, 53]. Nevertheless, some patients may require low doses of corticosteroids for several years [54]. It has been suggested that measuring the levels of interleukin-6 after four weeks of therapy is useful to identify patients with a more severe disease. The adoption of a combination of low dose methotrexate and azathioprine (resp., 5–7.5 mg/week and 50–75 mg/day) can be associated with corticosteroids in patients with an inadequate response to the standard therapy or in order to reduce the dose of glucocorticoids, with contrasting results [55, 56]. The use of anti-TNF agents is limited to the experiences of case series particularly in relapsing disease [57, 58], *α*-TNF inhibition with etanercept (25 mg subcutaneously twice weekly), seems to be more successful in discontinuing prednisone therapy and required a significantly lower cumulative prednisone dose after 12 months of treatment [59]. Although not definitive, these findings suggest that etanercept suppresses disease activity in refractory GCA [60]. A further *α*-TNF blocker evaluated in this setting is adalimumab, with encouraging results [61].

Anakinra, an anti-IL-1 whose effectiveness was tested for rheumatoid arthritis, Still's disease, and Muckle-Wells syndrome, seems to be a valid alternative in some cases of GCA, resistant or refractory to the steroid methotrexate or with azathioprine treatments, or even with etanercept, even if its use in GCA is limited to case reports [62].

Recently the role of an IL-6 blockade, tocilizumab, administered by infusion with a dose of 8 mg/kg, has been emphasized as a valid option in the treatment of GCAs and Takayasu's arteritis (TA) [49]. IL-6 inhibitor allowed not only a remarkable reduction of the clinical symptoms, but also a remission of the inflammation of the vascular wall, as shown in the magnetic resonance and the reduction of RCP and ERS.

Other available drugs are rituximab, an anti-CD20 monoclonal antibody [63], and abatacept, a recombinant fusion protein that modulates CD28-mediated T cell costimulation [64].

In Behcet's disease treatment of autoimmune condition reduces the risk of thrombosis.

## 3. Prognosis

Giant cell arteritis generally presents a good prognosis [8]; the mortality rate in patients with PM and GCA is similar to that expected for the age-matched general population; the response to the corticosteroid therapy is usually rapid after a few days. About 30–40% of the patients, especially during the first two years, present spontaneous exacerbations of the disease notwithstanding the steroid therapy. Recent studies showed that 92% of the visual deficiencies coming before therapy have begun and if the therapy begins within 24 hours after the onset of the symptoms, 58% of the patients find an improvement in their eyesight versus only 6% of the cases treated later [65]. The dissection of the aorta constitutes an important late complication of giant cell arteritis, worsening the generally good prognosis [29]. Recurrence or flares in GCA are frequent; the incidence of recurrence is about 40% [8, 27]. A retrospective study of GCA observed that the incidence of aortic aneurysm and dissection increased after 5 years from diagnosis; these patients presented a worse prognosis, with a higher cardiovascular and pulmonary death as compared to general population [66]. In a retrospective analysis of 174 patients with biopsy-proven GCA the presence of anemia at the time of diagnosis was the best predictor of relapses or recurrences of GCA (odds ratio, 2.17; 95% confidence interval, 1.02–4.62; *P* = 0.04). Seventy-one subjects (40.8%) experienced relapses or recurrences of the disease during follow-up; these patients did not show clinical differences when compared with the remaining biopsy-proven GCA patients. However, the total duration of corticosteroid therapy was significantly longer, suggesting that chronic inflammatory response manifested by anemia may predict the development of disease recurrences [67].

## 4. Conclusions

Thromboembolic complications in Horton's vasculitis represent an important feature of the disease. The advanced age of these patients together with the concomitant risk factors such as hypercholesterolemia, diabetes mellitus, arterial hypertension, cigarette smoking, previous cardiovascular events, obesity, and familial history of thrombosis may contribute to increasing of the thrombotic risk.

The efficacy of antithrombotic therapy in preventing cardiovascular events in these patients is still being evaluated; the presence of traditional cardiovascular risk factors may influence the choice of administering an antithrombotic therapy.

The steroids constitute the gold standard for the induction of the remission of the disease and have gradually been enriched with the adoption of drugs capable of having an impact on the molecules on the basis of the disease, such as the anti-TNF and the IL-6 blockade agents. Further clinical trials, finalized to a greater extent, may help improve the treatment of giant cell arteritis.

## Figures and Tables

**Table 1:** Clinical characteristics of GCA; percentage of patients presenting the indicated features.

Symptoms	Signs
Severe headaches (75%)	Temporal artery tenderness
Jaw claudication (50%)	Impaired or absent temporal pulse
Visual loss (temporary or permanent) (20%)	Fever
Tongue pain/dysphagia/impaired swallowing	Extraocular muscle weakness
Dyspnoea/cough (10%)	Neurological symptoms (30%), of which 15% are neuropathy
Vestibular/cochlear dysfunction	Raynaud's phenomenon
Muscle-skeletal pain (25%)	Arthritis/synovitis

**Table 2:** Criteria for the diagnosis of giant cell arteritis (American College of Rheumatology, 1990) (mod. from Hunder et al. [31]).

Criteria	Definition
Age	Onset of the symptoms at age 50 or older
Recent onset of severe headache	Onset of a new type of pain located in the head
Anomalies of the temporal artery	Pain in the palpation of the temporal artery or reduced pulsatility, not connected to atherosclerosis of the cervical arteries
Increase of ESR	ESR > 40 mm/h
Typical abnormalities of the biopsy of the temporal artery	Vasculitis typically characterized by an infiltrate of mononuclear cells or inflammation of granuloma with multinuclear giant cells
